# The Effects of Postprandial Walking on the Glucose Response after Meals with Different Characteristics

**DOI:** 10.3390/nu14051080

**Published:** 2022-03-04

**Authors:** Alessio Bellini, Andrea Nicolò, Ilenia Bazzucchi, Massimo Sacchetti

**Affiliations:** Department of Movement, Human and Health Sciences, University of Rome “Foro Italico”, Piazza Lauro De Bosis 6, 00135 Rome, Italy; alessiobellini1@gmail.com (A.B.); andrea.nicolo@uniroma4.it (A.N.); ilenia.bazzucchi@uniroma4.it (I.B.)

**Keywords:** post-meal glycemia, postprandial exercise, breakfast exercise, post-meal exercise

## Abstract

We evaluated the effect of postprandial walking on the post-meal glycemic response after meals with different characteristics. Twenty-one healthy young volunteers participated in one of two randomized repeated measures studies. Study 1 (10 participants) assessed the effects of 30 min of brisk walking after meals with different carbohydrate (CHO) content (0.75 or 1.5 g of CHO per kg/body weight). Study 2 (11 participants) evaluated the effects of 30 min of brisk walking after consuming a mixed meal or a CHO drink matched for absolute CHO content (75 g). Postprandial brisk walking substantially reduced (*p* < 0.009) the glucose peak in both studies, with no significant differences across conditions. When evaluating the glycemic response throughout the two hours post-meal, postprandial walking was more effective after consuming a lower CHO content (Study 1), and similarly effective after a mixed meal or a CHO drink (Study 2), although higher glucose values were observed when consuming the CHO drink. Our findings show that a 30 min postprandial brisk walking session improves the glycemic response after meals with different CHO content and macronutrient composition, with implications for postprandial exercise prescription in daily life scenarios.

## 1. Introduction

Elevated postprandial blood glucose concentration and large glycemic excursions have been identified as better predictors of cardiometabolic disorders than fasting hyperglycemia in both healthy individuals and diabetic patients [1]. Indeed, exaggerated blood glucose spikes lead to a higher increase in oxidative stress [2], endothelial dysfunction [2,3], proinflammatory factors levels [4], and in the risk of developing cardiovascular pathologies [5] than fasting hyperglycemia. Exercise and nutrition have a fundamental role in the management of excessive elevation of post-meal glycemia [6], and previous studies have shown that postprandial exercise, also in relation to the modality it is administered, is effective in improving the glycemic response to a standard meal [6,7,8,9,10,11]. However, it is currently unclear if the prescription of postprandial exercise should take into account the characteristics of the meal and how.

Among the exercise parameters, exercise timing has a key role in improving post-meal glycemic control [7,8,12,13]. Previous studies have widely demonstrated that exercising in the period immediately after the meal provides a greater reduction in the post-meal glycemic peak compared with pre-meal exercise, especially when exercise starts before reaching peak glucose levels [8,14]. Other parameters have a lower impact on the glucose response to a meal compared with exercise timing. For instance, the modulation of exercise intensity and duration does not lead to substantial variations in postprandial glucose concentration in healthy individuals [8,15]. Likewise, aerobic, resistance, or combined exercises are similarly effective in improving the post-meal glycemic response [7,8]. Hence, moderate-intensity walking appears to be a feasible exercise option for everyone as it can easily be performed without the need for any equipment and supervision of an exercise specialist. Importantly, postprandial walking has been proven effective for improving glycemic response to different meals of the day (i.e., breakfast, lunch, and dinner) both in healthy and diabetic individuals [8,11,14,16,17,18,19].

Nutrition is another important factor to consider when attempting to improve the glycemic response to a meal. While the meal characteristics may widely vary in daily life, the studies assessing the effects of postprandial exercise on glycemia have rarely attempted to evaluate the effects of exercise after different mixed meals. A large body of research focused on the effects of postprandial exercise after the consumption of an oral glucose tolerance test (OGTT), thus inducing a different glycemic response compared with that of a mixed meal [20], which better resembles what is often consumed in daily life. When the use of a mixed meal was implemented, it was consistently shown that 30 min of step cadence paced moderate-intensity walking is effective in improving the glycemic response to a meal providing 1 g of CHO per kg of body weight [8]. However, it is currently unclear how the effectiveness of a typical postprandial walking session (e.g., 30 min of brisk walking) would change in relation to meals with different CHO content and composition.

Indeed, among the nutritional factors affecting the glycemic response to a meal, the amount of carbohydrate (CHO) provided with the meal plays an important role. Previous studies have documented a higher glycemic response with the increase in the meal CHO content [21]. The macronutrient characteristics of the meal may also have a relevant influence on the postprandial glucose response. Indeed, high-protein and/or high-fat meals induce a significant reduction in the postprandial glucose response [22,23,24,25,26]. In addition, whether a meal is solid or liquid should also be considered for the impact on the postprandial glycemic response, with higher glycemic excursions observed after a liquid meal compared with a solid one [27,28].

Therefore, we performed two studies aiming to determine the effects of postprandial walking on the glycemic response to meals with different characteristics. Study 1 evaluated the effects of exercise after mixed meals with different CHO content, while Study 2 assessed the effects of exercise after the consumption of meals with a different macronutrient composition (e.g., mixed meal vs. CHO drink (i.e., OGTT)), but the same amount of CHO. Collectively, findings from the two studies were expected to assess the efficacy of 30 min of postprandial brisk walking when varying some characteristics of the meal, with potential implications for implementing this simple exercise strategy in daily life.

## 2. Materials and Methods

### 2.1. Participants

Twenty-three healthy, physically active (reaching the minimum amount of physical activity recommended by the World Health Organization guidelines [29]), young and healthy adults (20–35 years old) volunteered to participate in this investigation. In total, twenty-one individuals completed all the experimental protocols of one of the two studies. More detailed information on participants’ characteristics is reported in Table 1. The studies were conducted in accordance with the Declaration of Helsinki and ethical approval was provided by the Local Ethical Committee (52/2020, 11 June 2020). Written informed consent was obtained from all the volunteers involved in the study.

### 2.2. Study Overview

**Figure 1:**
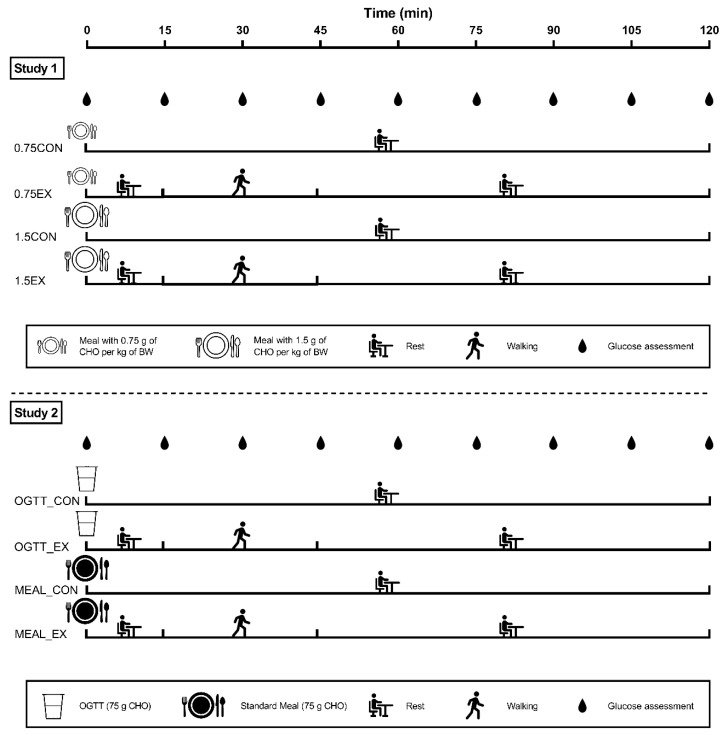
Graphic representation of the two studies. In Study 1, participants consumed a meal containing 0.75 g of carbohydrates (CHO) per kg of body weight (BW) (0.75CON and 0.75EX), or 1.5 g of CHO per kg of BW (1.5CON and 1.5EX). In Study 2, participants consumed 75 g of CHO alone dissolved in water (OGTT_CON and OGTT_EX) or 75 g of CHO combined with protein and fat in a solid mixed meal (MEAL_CON and MEAL_EX). For both studies, after each meal participants performed 30 min of walking started 15 min after the beginning of the meal (0.75EX, 1.5EX, OGTT_EX, and MEAL_EX) or remained seated for the whole experimental period (0.75CON, 1.5CON, OGTT_CON, and MEAL_CON). After the 30 min of walking, participants remained seated until the end of the experimental period.

Volunteers performed one of the two repeated measures studies with four experimental trials per study. At the beginning of each study, a familiarization visit was performed. Subsequently, participants performed four experimental visits in a randomized order, lasting 2 h each. At least seventy-two hours of rest were considered between visits in order to avoid any residual effect of exercise [30]. In Study 1, participants consumed a breakfast differing in CHO content (i.e., 0.75 vs. 1.5 g of CHO per kg of body weight), while in Study 2 participants consumed a breakfast differing in macronutrient composition (i.e., CHO drink vs. mixed meal) with the same amount of CHO (i.e., 75 g). In each study and meal condition, after breakfast participants performed 30 min of moderate-intensity walking or remained seated for the whole experimental period. A schematic representation of the design of the two studies is shown in Figure 1.

### 2.3. Familiarization

Before the beginning of each study, volunteers participated in a familiarization session, during which all the experimental procedures adopted were explained. Participants were requested to avoid moderate-to-vigorous physical activity for the 48 h preceding each experimental visit and to abstain from caffeine and alcohol consumption since the evening before the visit. Participants were also requested to register activities performed during the 48 h and food consumed during the 24 h before the first experimental visit and to replicate them before the remaining three visits.

### 2.4. Study 1—The Effects of Postprandial Exercise on Glycemia after Consuming Mixed Meals with Different CHO Content

Ten participants were included in this study and completed all the visits (Table 1). Participants attended the laboratory at 08.00 a.m. after an overnight fasting (>10 h of fasting). At 09.00 a.m., participants consumed one of two meals high in CHO content, containing 0.75 g (0.75CON and 0.75EX) or 1.5 g (1.5CON and 1.5EX) of CHO per kg of body weight. The meal consisted of partially skimmed milk, rusks, and jam (Table 2). Participants were given 10 min to finish their meal. After each meal, participants remained seated until the end of the experimental session (0.75CON and 1.5CON) or performed 30 min of walking starting 15 min after the beginning of the meal (0.75EX and 1.5EX), as shown in Figure 1.

### 2.5. Study 2—The Effects of Postprandial Exercise on Glycemia after Consuming Meals with Different Macronutrient Composition

Eleven participants were included in this study and completed all the visits (Table 1). As for Study 1, participants attended the laboratory at 08.00 a.m. after an overnight fasting (>10 h of fasting), and at 09.00 a.m. consumed 75 g of glucose (Yamamoto Nutrition, Italy) dissolved in 300 mL of water (as commonly done for the OGTT) or a mixed meal, providing the same amount of CHO. The mixed meal consisted of 280 mL partially skimmed milk, 44 g rusks, and 50 g jam (MEAL_CON and MEAL_EX). Detailed information on the macronutrient composition of the two meals is reported in Table 2. Participants were given 10 min to finish their meal. As for Study 1, after the meal, participants remained seated for the whole experimental period (OGTT_CON and MEAL_CON) or performed 30 min of exercise starting 15 min after the beginning of the meal (OGTT_EX and MEAL_EX) (Figure 1).

### 2.6. Exercise and Resting Time

Participants were invited to remain seated throughout all the visits of the two studies, except for the 30 min of exercise or for using services. While sitting they were allowed to read or use the PC, but they were asked to replicate their actions during all the visits.

The exercise was the same for the four exercise visits of the two studies (i.e., 0.75EX, 1.5EX, OGTT_EX, and MEAL_EX) (Figure 1). Specifically, it consisted of 30 min of walking, started 15 min after the beginning of the meal (09.15 a.m.), at 120 steps per minute, rhythmically established through a digital metronome (Soundbrenner, Berlin, Germany). Participants walked alone on an indoor track. The use of step cadence has been previously proposed as a valid estimate of the metabolic cost of walking [31,32]. In addition, the step cadence may also be a practical mode for prescribing exercise intensity in real life. At the end of the exercise session, participants remained seated for the remaining experimental time.

### 2.7. Glycemic Assessment

Capillary blood glucose measures were regularly collected and analyzed by using reactive strips and a glucometer (Contour^®^Next, Bayer HealthCare S.p.A., Milan, Italy). Two measures were collected and the average of the two was considered. When a difference greater than 10% between the two measures was found, a third measure was collected. Glycemia was assessed at fasting and every 15 min after the meal, until the end of the visit (Figure 1). Before each measure was performed in any of the visits, participants washed their hands in order to avoid possible alterations of the measure related to external factors.

### 2.8. Rating of Perceived Exertion

In both studies, during the exercise conditions (i.e., 0.75EX and 1.5EX, in Study 1, and OGTT_EX and MEAL_EX, in Study 2), perceived exertion was evaluated using the rating of perceived exertion (RPE) Borg’s 6–20 scale every 15 min during the 30 min walking (i.e., 30 and 45 min from the beginning of the meal).

### 2.9. Statistical Analysis

Statistical analysis was performed using the software IBM SPSS statistics version 23.0 (SPSS Inc., Chicago, IL, USA). The analyses performed were identical in both studies for all variables. Data normality was checked using the Shapiro–Wilk test. The glycemic time course was compared across conditions using a two-way repeated-measures ANOVA (condition × time). In the case of significant interactions, the simple main effect of condition at each time point was analyzed using a one-way repeated-measures ANOVA. Mean blood glucose concentration at 0–120 min was also calculated and analyzed using a one-way repeated-measures ANOVA. The time-averaged positive incremental area under the curve (iAUC) was calculated at 0–60, 60–120, and 0–120 min [33]. A one-way repeated measures ANOVA was used to analyze differences between conditions for positive iAUC. RPE values were compared across conditions using a two-way repeated-measures ANOVA (condition × time).

The Greenhouse–Geisser or the Huynh–Feldt corrections were used for adjusting the degrees of freedom of the within-subject comparisons for ε < 0.75 and ε > 0.75, respectively. In the case of significant differences, the least significant differences (LSD) correction was used for the analysis of multiple comparisons. For all statistical tests, the level of significance was set at 0.05. Partial eta squared (η_p_^2^) effect sizes were determined, considering η_p_^2^ ≥ 0.01 as small, η_p_^2^ ≥ 0.059 as medium, and η_p_^2^ ≥ 0.138 as large [34]. Values are reported as mean (±SD) in tables and in the text, and as mean (±SEM) in figures.

## 3. Results

### 3.1. Study 1—The Effects of Postprandial Exercise on Glycemia after Consuming Mixed Meals with Different CHO Content

**Figure 2:**
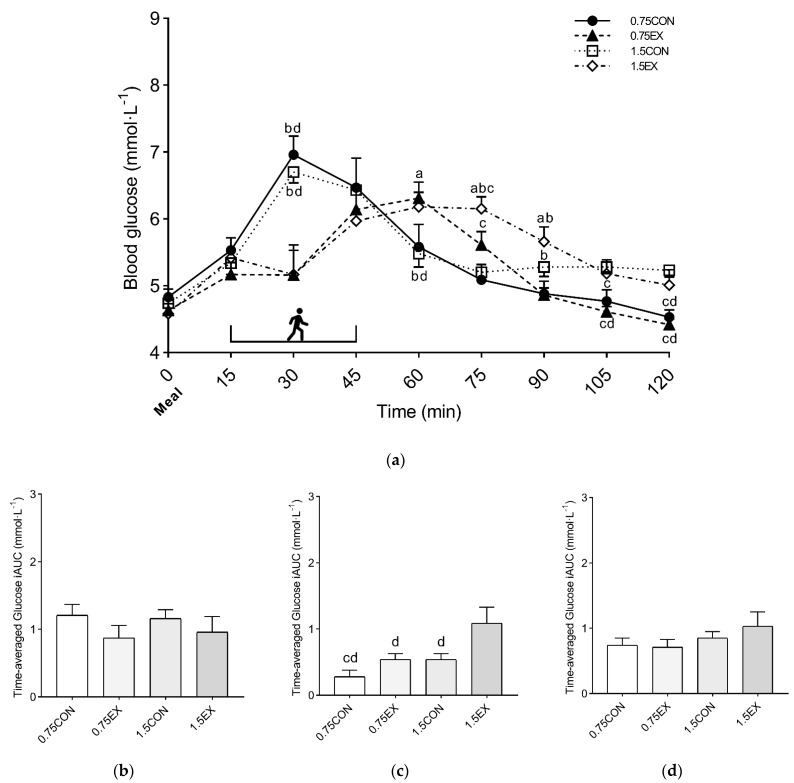
Glycemic time course (**a**) and time-averaged positive iAUC at 0–60 min (**b**), 60–120 min (**c**), and 0–120 min (**d**) of Study 1. Symbols: a, *p* < 0.05 vs. 0.75CON; b, *p* < 0.05 vs. 0.75EX; c, *p* < 0.05 vs. 1.5CON; and d, *p* < 0.05 vs. 1.5EX. The half-box represents the exercise sessions. Values are reported as mean (±SEM).

A significant interaction (condition × time) was found when comparing the glycemic time course between conditions (*p* < 0.001, η_p_^2^ = 0.506). The exercise conditions significantly (*p* < 0.009) reduced the glycemic peak at 30 min (0.75EX, 5.16 ± 1.42 mmol·L^−1^ and 1.5EX, 5.17 ± 1.15 mmol·L^−1^) compared with the control conditions (0.75CON, 6.96 ± 0.89 mmol·L^−1^ and 1.5CON, 6.70 ± 0.52 mmol·L^−1^). At 120 min, 0.75CON (4.53 ± 0.36 mmol·L^−1^) and 0.75EX (4.42 ± 0.25 mmol·L^−1^) showed significantly lower values compared with 1.5CON (5.23 ± 0.30 mmol·L^−1^) and 1.5EX (5.01 ± 0.71 mmol·L^−1^) (*p* < 0.041). Detailed information on the simple main effect of conditions at each time point is reported in Figure 2.

The analysis of the time-averaged positive iAUC did not show significant differences between conditions either at 0–60 min (0.75CON, 1.21 ± 0.49 mmol·L^−1^; 0.75EX, 0.87 ± 0.59 mmol·L^−1^; 1.5CON, 1.16 ± 0.41 mmol·L^−1^; and 1.5EX, 0.96 ± 0.71 mmol·L^−1^) or 0–120 min (0.75CON, 0.74 ± 0.11 mmol·L^−1^; 0.75EX, 0.71 ± 0.40 mmol·L^−1^; 1.5CON, 0.85 ± 0.30 mmol·L^−1^; and 1.5EX, 1.03 ± 0.69 mmol·L^−1^). Conversely, significantly higher values were found at 60–120 min for 1.5EX (1.09 ± 0.24 mmol·L^−1^) compared with 0.75CON (0.28 ± 0.10 mmol·L^−1^), 0.75EX (0.54 ± 0.09 mmol·L^−1^), and 1.5EX (0.54 ± 0.09 mmol·L^−1^) (*p* < 0.03). In addition, 1.5CON showed significantly higher values compared with 0.75CON (*p* = 0.048) (Figure 2), while a statistical trend (*p* = 0.063) was found when comparing 0.75CON with 0.75EX.

No significant differences were found between conditions for 0–120 mean blood glucose concentration (0.75CON, 5.40 ± 0.57 mmol·L^−1^; 0.75EX, 5.21 ± 0.46 mmol·L^−1^; 1.5CON, 5.52 ± 0.30 mmol·L^−1^; and 1.5EX, 5.48 ± 0.54 mmol·L^−1^).

No significant differences were found across conditions for RPE either at 30 (0.75EX, 9.95 ± 2.03; 1.5EX 10.20 ± 1.30) or 45 min (0.75EX, 10.25 ± 1.99; 1.5EX 10.45 ± 1.38).

### 3.2. Study 2—The Effects of Postprandial Exercise on Glycemia after Consuming Meals with Different Macronutrient Composition

**Figure 3:**
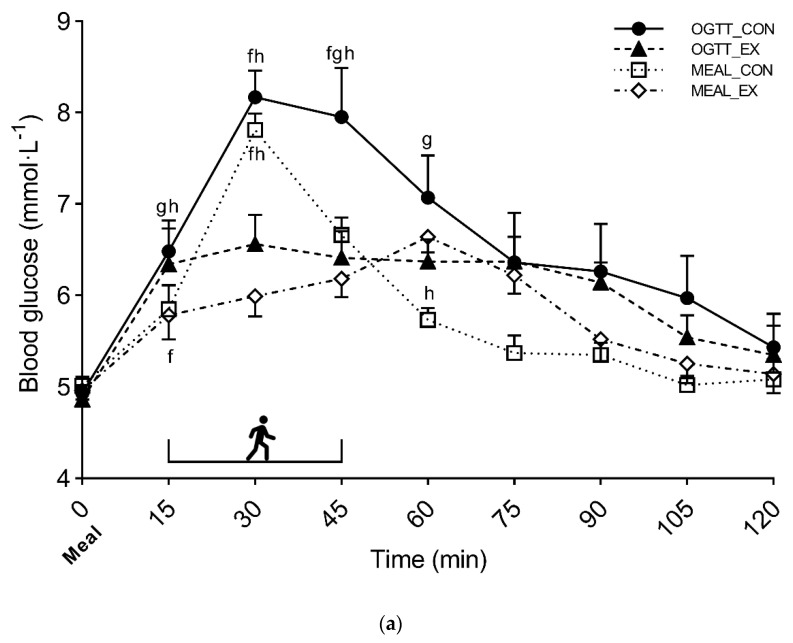

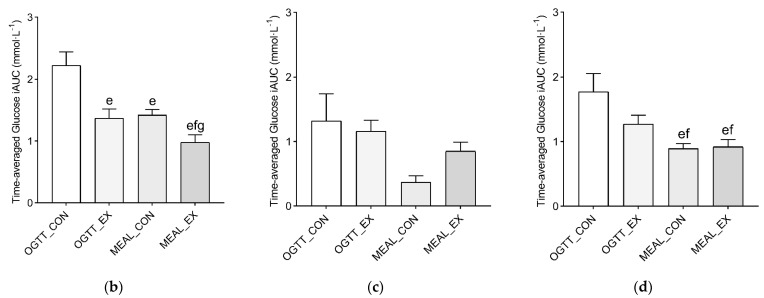
Glycemic time course (**a**) and time-averaged positive iAUC at 0–60 min (**b**), 60–120 min (**c**), and 0–120 min (**d**) of Study 2. Symbols: e, *p* < 0.05 vs. OGTT_CON; f, *p* < 0.05 vs. OGTT_EX; g, *p* < 0.05 vs. MEAL_CON; and h, *p* < 0.05 vs. MEAL_EX. The half-box represents the exercise sessions. Values are reported as mean (±SEM).

A significant interaction (condition × time) was found when comparing the glycemic time course between conditions (*p* < 0.001, η_p_^2^ = 0.381). The exercise conditions significantly (*p* < 0.004) reduced the glycemic peak at 30 min (OGTT_EX, 6.56 ± 1.07 mmol·L^−1^ and MEAL_EX, 5.99 ± 0.74 mmol·L^−1^) compared with the control conditions (OGTT_CON, 8.17 ± 0.95 mmol·L^−1^ and MEAL_CON, 7.81 ± 0.59 mmol·L^−1^). At 45 min, OGTT_CON (7.95 ± 1.79 mmol·L^−1^) showed significant higher glucose values compared with OGTT_EX (6.41 ± 0.95 mmol·L^−1^), MEAL_CON (6.66 ± 0.63 mmol·L^−1^), and MEAL_EX (6.18 ± 0.65 mmol·L^−1^) (*p* < 0.028). Detailed information on the simple main effect of conditions at each time point is reported in Figure 3.

The time-averaged positive glucose iAUC showed significantly higher values at 0–60 min for OGTT_CON (2.22 ± 0.74 mmol·L^−1^) compared with OGTT_EX (1.37 ± 0.51 mmol·L^−1^), MEAL_CON (1.42 ± 0.32 mmol·L^−1^) and MEAL_EX (0.98 ± 0.38 mmol·L^−1^) (*p* < 0.005). In addition, MEAL_EX showed significantly lower glucose values compared with OGTT_EX and MEAL CON (*p* < 0.034). Significantly lower values were also found at 0–120 min in MEAL_CON (0.89 ± 0.27 mmol·L^−1^) and MEAL_EX (0.92 ± 0.38 mmol·L^−1^) compared with OGTT_CON (1.77 ± 0.94 mmol·L^−1^) and OGTT_EX (1.27 ± 0.48 mmol·L^−1^) (*p* < 0.011 and *p* < 0.025, respectively). A statistical trend was found for positive iAUC at 60–120 min (*p* = 0.065) (Figure 3).

Mean blood glucose concentration (0–120 min) was significantly lower in MEAL_CON (5.76 ± 0.28 mmol·L^−1^) and MEAL_EX (5.74 ± 0.43 mmol·L^−1^) compared with OGTT_CON (6.51 ± 1.05 mmol·L^−1^) (*p* < 0.036). In addition, a statistical trend was observed between MEAL_EX and OGTT_EX (5.99 ± 0.54 mmol·L^−1^) (*p* = 0.053).

No significant differences were found across conditions for RPE either at 30 (OGTT_EX, 10.82 ± 0.87; MEAL_EX, 11.27 ± 1.13) and 45 min (OGTT_EX, 11.18 ± 1.08; MEAL_EX, 11.68 ± 1.15).

## 4. Discussion

Improving the post-meal glycemic response is important for reducing cardiometabolic disorders both in healthy individuals and in patients with diabetes [1,7,12,35]. While 30 min of postprandial walking has proven to be effective in attenuating the glycemic response after a standard meal [8], less is known on its efficacy in relation to the amount of CHO provided with the meal or to its macronutrient composition. Hence, we performed two studies collectively showing that 30 min of postprandial walking is:

(i) Effective in reducing the glucose peak both when increasing the CHO content of a mixed meal and when consuming a CHO drink.
(ii) Less effective in improving the total glycemic response two hours after the meal when the CHO content of a mixed meal is relatively high.

These findings have implications for planning exercise sessions aimed at improving the postprandial glycemic response after meals with different characteristics.

The results of our two studies showed that performing 30 min of walking exercise, started immediately after the meal, effectively attenuated the glucose peak after meals with different CHO quantities and compositions. Study 1 showed that the post-meal glucose peak was similarly reduced by exercise when the amount of CHO was either 0.75 or 1.5 g per kg of body weight. This suggests that moderate-intensity exercise has an important impact on the glucose peak after the consumption of a meal with a relatively high amount of CHO in healthy individuals. Likewise, a similar reduction in the glucose peak was observed when participants performed moderate walking either after consuming an OGTT or a mixed meal, even though the latter had a greater energy intake. This attenuation of the post-meal glucose peak has implications for the reduction in the cardiometabolic disorders associated with it, as suggested by the reduction in the levels of markers associated with oxidative stress [36]. Our results extend previous findings on the effectiveness of postprandial exercise in reducing the post-meal glycemic peak [8,14,16] to meals characterized by different CHO levels and contents of macronutrients. This constitutes a step forward for suggesting the implementation of a 30 min postprandial moderate walk in daily life scenarios, where the meal content and composition may substantially vary.

Although we found a similar reduction in the glycemic peak after consuming mixed meals with two different amounts of CHO (Study 1), the CHO content of the meal moderated the effect of postprandial exercise on the glycemic response when considering the first two hours after the meal. Indeed, a substantial glycemic rebound was found after exercise when participants consumed 1.5 g of CHO per kg of body weight, with higher glycemic values observed throughout the second hour post-meal when compared with the 0.75EX condition. The extent of the glycemic rebound in the 1.5EX condition can be further appreciated when considering that the time-averaged glucose iAUC value was significantly higher than that of the other three conditions (Figure 2c). Although we did not investigate the mechanisms underlying this effect, higher glycemic values may be related to the longer release of the meal-derived glucose from the gastrointestinal system [37]. These findings suggest that when consuming a meal with a high CHO content, 30 min of postprandial continuous walking started early after the meal are sufficient to elicit a marked attenuation of the early glucose response, while it is less effective in the late postprandial phase. In this context, other exercise strategies should be considered to improve the glucose response over the entire post-meal period. For instance, previous evidence has shown the effectiveness of spreading the exercise session into shorter activity breaks over the entire postprandial period for preventing the glycemic rebound and improving the glucose iAUC response [8,38,39,40,41]. Further studies should investigate whether activity breaks may also provide a relevant stimulus for reducing the glucose levels over the two hours even when the CHO content of a meal is high.

The comparison between the effects of postprandial exercise after a mixed meal or a CHO drink matched for CHO content allowed us to gain further insight into the glycemic response both from a methodological and practical perspective. While an OGTT is often used in research to evaluate the effects of postprandial exercise on the glycemic response, it does not generally reproduce real-life conditions, hence impacting on the applicability of the study findings. We have compared the CHO drink with a mixed meal with the same absolute content of CHO, which is similar to the standard meal that we have previously used [8]. As expected, the CHO drink consumption results in a more rapid increase in glycemia and in higher glycemic values throughout the first two hours after the meal compared with the glycemic response observed after the mixed meal. The presence of fat and protein in the mixed meal may explain this difference between the OGTT and the mixed meal. Indeed, several studies have shown that the presence of these macronutrients may delay gastric emptying and attenuate the glycemic response [22,25,26]. Similarly, the greater energy intake and the semi-solid composition of the mixed meal may also have contributed to delaying gastric emptying compared with the OGTT [28]. It is also conceivable that the greater insulin secretion that usually occurs after the consumption of a mixed meal may have contributed to improving the glycemic response in that experimental condition [23]. These findings suggest caution when assessing the effect of postprandial exercise with an OGTT test, as the factors determining glycemic control may differ compared with a mixed meal, as also shown in previous studies [20,28]. From a practical perspective, this comparison allowed us to show that exercise is effective even when a CHO-only drink is consumed.

While we did not attempt to investigate the mechanisms underlying the effect of postprandial exercise on glycemic control after meals with different characteristics, the systematic manipulation of the meal has the potential to shed some light on this issue. Indeed, different meals may have different effects on the factors affecting the rate of blood glucose appearance and disappearance. This applies, for instance, to the glucose release from the gastrointestinal system [37,42,43], and to insulin-dependent glucose uptake [44]. Hence, the manipulation of the meal should be accompanied by the assessment of relevant hormonal (e.g., insulin levels) and physiological responses in future studies. An improved understanding of the mechanisms underlying the interaction between meal and exercise would further refine the prescription of postprandial exercise.

## 5. Conclusions

We conducted two studies assessing the efficacy of 30 min of postprandial brisk walking performed 15 min after meals with different CHO content or macronutrient composition. Study 1 showed that exercise similarly reduces the glycemic peak after mixed meals containing either 0.75 or 1.5 g of CHO per kg of body weight, while it is less effective in improving the glycemic response throughout the first two hours post meal when the amount of CHO in the meal is relatively higher. Study 2 showed that the glycemic response differs both in time course and absolute values after an OGTT or a mixed meal matched for CHO content, with similar improvements when postprandial exercise is performed.

Collectively, our findings show that 30 min of postprandial brisk walking is effective in improving the glycemic response after meals with different CHO content or macronutrient composition. These results support the implementation of walking among the tools for improving glycemic control in everyday life scenarios, where the content and composition of a meal may vary substantially. This study was performed in young, healthy individuals, and further studies are required to evaluate whether similar responses may occur in older, unfit, or individuals with metabolic disorders.

## Figures and Tables

**Table 1:** Participants’ characteristics of the two studies.

	Study 1	Study 2
Sample size (M/F)	10 (5/5)	11 (5/6)
Age (years)	25 ± 3	25 ± 2
Weight (kg)	66 ± 9	68 ± 10
Height (m)	1.70 ± 0.09	1.74 ± 0.13
BMI (kg/m^2^)	22.9 ± 2.6	22.5 ± 2.3

Abbreviations: M, male; F, female; BMI, body mass index. Data are expressed as mean ± SD.

**Table 2:** Meal composition in the two studies.

	Study 1		Study 2	
	Meal 1	Meal 2	Meal 1	Meal 2
Energy intake (kcal)	276.20 ± 29.97	551.73 ± 62.57	297.00 ± 0.00	421.84 ± 0.00
CHO (g)	50.57 ± 5.76	100.77 ± 11.34	75.00 ± 0.00	75.00 ± 0.00
Protein (g)	8.57 ± 8.51	17.22 ± 4.32	0 ± 0.00	14.50 ± 0.00
Fat (g)	4.12 ± 1.23	8.28 ± 2.46	0 ± 0.00	6.78 ± 0.00
CHO (%)	73.79 ± 6.17	73.66 ± 6.05	100.00 ± 0.00	71.38 ± 0.00
Protein (%)	12.38 ± 12.37	12.45 ± 12.40	0 ± 0.00	13.82 ± 0.00
Fat (%)	13.35 ± 3.48	13.42 ± 3.42	0 ± 0.00	14.51 ± 0.00

In Study 1, Meal 1 consists in 0.75 g of carbohydrates (CHO) per kg of body weight and Meal 2 in 1.5 g of CHO per kg of body weight. In Study 2, Meal 1 consists in 75 g of CHO dissolved in water and Meal 2 in 75 g consumed in a mixed meal along with protein and fat. Data are expressed as mean ± SD.

## Data Availability

The data presented in this study are available on request from the corresponding author.
